# Graves’ Disease Mimicking Orthostatic Dysregulation in an Adolescent Girl: Findings on Active Standing Tests

**DOI:** 10.7759/cureus.117563

**Published:** 2026-10-06

**Authors:** Takuya Itayagoshi, Shoichiro Kanda, Haruka Shimizu, Moe Yoshimura, Ikuma Musha, Yuko Akioka, Toru Kikuchi

**Affiliations:** 1 Pediatrics, Saitama Medical University, Moroyama, JPN

**Keywords:** active standing test (as test), adolescent medicine, graves' disease, orthostatic dysregulation, postural orthostatic tachycardia syndrome (pots)

## Abstract

Orthostatic dysregulation (OD) is common during adolescence and may present with dizziness, palpitations, fatigue, and difficulty getting up in the morning. These symptoms overlap with those of Graves’ disease, making differentiation challenging in some patients. A 14-year-old girl presented with headache, dizziness, palpitations, and difficulty getting up in the morning, with worsening symptoms during standing and walking. An active standing test reproduced her symptoms, including tachycardia, lower-extremity tremor, marked sweating, and nausea, and she was initially considered to have OD. Her resting heart rate was already elevated, and marked orthostatic tachycardia resembling postural orthostatic tachycardia syndrome (POTS) was observed during the test. However, thyroid function tests revealed severe hyperthyroidism, and positive thyroid-stimulating hormone receptor antibodies led to a diagnosis of Graves’ disease. Following treatment with methimazole, potassium iodide, and propranolol, her symptoms resolved. Propranolol was discontinued after one month. Repeat orthostatic testing after improvement of thyroid function no longer reproduced her symptoms or marked tachycardia. Graves’ disease may therefore mimic not only the clinical symptoms of OD but also POTS-like orthostatic tachycardia during orthostatic testing. Thyroid dysfunction may warrant consideration in patients with OD-like symptoms, particularly when accompanied by resting tachycardia, tremor, increased sweating, or weight loss.

## Introduction

Orthostatic dysregulation (OD) is a condition characterized by symptoms such as lightheadedness, dizziness, marked fatigue, particularly in the morning, headache, and palpitations, resulting from impaired or dysfunctional regulatory mechanisms, including the autonomic nervous system, that are involved in cardiovascular regulation during orthostasis. OD is commonly observed during adolescence [1]. Postural orthostatic tachycardia syndrome (POTS) is an important form of orthostatic intolerance characterized by excessive heart rate elevation during standing accompanied by orthostatic symptoms [2]. Graves’ disease also frequently develops after puberty, particularly in females, and may cause palpitations, tachycardia, sweating, fatigue, weight loss, and tremor [3,4]. Because of the considerable overlap in clinical symptoms between these two conditions, Graves’ disease may mimic OD, making it difficult to distinguish between them; indeed, cases of Graves’ disease presenting with orthostatic symptoms have been reported [5]. Furthermore, their treatment strategies differ substantially; therefore, accurate diagnosis is essential for determining the appropriate therapeutic approach. Here, we report a case in which a patient presented with symptoms suggestive of OD, and the symptoms were reproduced during the active standing test, initially leading to a diagnosis of OD. However, further evaluation ultimately resulted in a diagnosis of Graves’ disease.

## Case presentation

A 14-year-old girl presented with a one-month history of headache, nausea, palpitations, and dizziness. Her symptoms tended to be more pronounced in the morning, and she had difficulty getting out of bed in the morning and frequently felt unwell during the first half of the day. The symptoms were exacerbated by standing and walking. While walking approximately 30 minutes from her home to school, she occasionally developed malaise severe enough to make it difficult for her to continue walking. In addition, she had increasingly appeared pale during volleyball practice and had begun to experience palpitations and shortness of breath during exercise. She also occasionally reported experiencing “flickering” or “flashing” in her vision when playing the recorder at school.

Because of concern about anemia, she initially visited a local physician. Blood tests revealed mild abnormalities in liver function; however, no other significant abnormalities were identified. She was subsequently referred to our hospital for further evaluation.

She was born at 38 weeks and one day of gestation by cesarean section, with a birth weight of 2,844 g. Her past medical history was unremarkable, and she was not taking any medications. Her paternal grandmother had a history of Graves’ disease.

At the initial visit, her body temperature was 36.6 ℃, height was 154.3 cm, and weight was 50.6 kg. Laboratory tests revealed a mild elevation in alanine aminotransferase (ALT) to 43 U/L (reference range, 9-28.5 U/L) and a mildly decreased total cholesterol level of 116 mg/dL (reference range, 120-219 mg/dL), with no other significant abnormalities in the complete blood count, inflammatory markers, electrolytes, or renal function.

Because OD was suspected based on her symptoms, including headache, dizziness, and palpitations, an active standing test was performed [6]. Her resting heart rate was relatively high at 103-109 beats/min. Following standing, her heart rate increased to 123-147 beats/min. Approximately four to five minutes after standing, she developed tremors of the lower extremities, marked sweating, and nausea, and these symptoms gradually worsened. Her systolic blood pressure decreased from approximately 110 mmHg to around 100 mmHg. At approximately eight minutes after standing, the tremors of the lower extremities became pronounced, making it difficult for her to maintain an upright position. Although the decrease in blood pressure was mild, the active standing test reproduced her characteristic symptoms, supporting the initial clinical suspicion of OD. The marked orthostatic tachycardia also showed POTS-like features; however, the elevated resting heart rate suggested the possibility of an underlying cause of sinus tachycardia. Based on these findings and her clinical presentation, she was provisionally diagnosed with OD and was prescribed oral midodrine hydrochloride before being discharged home.

However, several hours later, thyroid function tests revealed markedly elevated free triiodothyronine (FT3) (>32.5 pg/mL; reference range, 2.3-4.0 pg/mL) and free thyroxine (FT4) (>7.77 ng/dL; reference range, 0.9-1.7 ng/dL) levels, with a suppressed thyroid-stimulating hormone (TSH) level (<0.01 μIU/mL; reference range, 0.5-5.0 μIU/mL), indicating hyperthyroidism. The patient and her family were contacted and instructed not to take midodrine hydrochloride and to return for further evaluation. At the follow-up visit, repeat physical examination revealed mild diffuse thyroid enlargement without exophthalmos or finger tremor. A more detailed history revealed heat intolerance, increased sweating, palpitations, occasional diarrhea, and a weight loss of approximately 3 kg over the preceding month. Additional laboratory testing revealed elevated thyroglobulin (521 ng/mL; reference range, 0-33.7 ng/mL), thyrotropin receptor antibody (TRAb) (15.6 IU/L; reference range, 0-1.9 IU/L), thyroid-stimulating antibody (TSAb) (369%; reference range, 0-109%), and thyroid peroxidase antibody (TPOAb) (53 IU/mL; reference range, 0-15 IU/mL) levels. Thyroglobulin antibody (TgAb) was within the reference range (21 IU/mL; reference range, 0-27 IU/mL). Thyroid ultrasonography demonstrated diffuse thyroid enlargement with mildly heterogeneous echogenicity and increased intrathyroidal vascularity, without apparent nodules. Based on the biochemical evidence of hyperthyroidism, positive TRAb and TSAb, and ultrasonographic findings, she was diagnosed with Graves’ disease.

Treatment was initiated with methimazole at 15 mg/day, potassium iodide at 50 mg/day, and propranolol at 30 mg/day. Following initiation of treatment, her symptoms, including palpitations, dizziness, and headache, rapidly improved, and the malaise associated with standing resolved. Her difficulty getting up in the morning and difficulty walking to school also resolved, allowing her to resume her daily activities and school life without limitations. Propranolol was discontinued one month after treatment initiation.

Three months after the initiation of treatment, while she remained on methimazole at 15 mg/day and potassium iodide at 50 mg/day, her thyroid function had improved, with an FT3 level of 4.09 pg/mL (reference range, 2.3-4.0 pg/mL), an FT4 level of 0.64 ng/dL (reference range, 0.9-1.7 ng/dL), and a TSH level of 0.11 μIU/mL (reference range, 0.5-5.0 μIU/mL). At the same time, the active standing test was repeated. Her resting heart rate was 56-68 beats/min and increased to a maximum of 81 beats/min after standing. Her blood pressure ranged from 106-113/52-64 mmHg at rest and from 93-113/55-81 mmHg after standing (Table 1). Unlike during the initial active standing test, she did not develop lower-extremity tremors, marked sweating, nausea, or other symptoms, and she was able to maintain an upright position without difficulty. Potassium iodide was subsequently discontinued, while methimazole at 15 mg/day was continued.

**Table 1:** Changes in Thyroid Function and Active Standing Test Findings Before and After Treatment Abbreviations: FT3, free triiodothyronine; FT4, free thyroxine; TSH, thyroid-stimulating hormone; BP, blood pressure.

Variable	At diagnosis	3 months after treatment	Reference range
Thyroid function			
FT3, pg/mL	>32.5	4.09	2.3-4.0
FT4, ng/dL	>7.77	0.64	0.9-1.7
TSH, μIU/mL	<0.01	0.11	0.5-5.0
Active standing test			
Resting heart rate, beats/min	103-109	56-68	‐
Heart rate during standing, beats/min	123-147	72-81	‐
Resting systolic BP, mmHg	115-121	106-113	‐
Systolic BP during standing, mmHg	104-123	93-113	‐
Lower-extremity tremor	Present from 4-5 min	Absent	‐
Marked sweating	Present from 4-5 min	Absent	‐
Nausea	Present from 4-5 min	Absent	‐
Unsteadiness while standing	Present from approximately 8 min	Absent	‐

## Discussion

The key feature of this case was that Graves’ disease not only mimicked the clinical symptoms of OD but also produced OD-like findings during the active standing test. The patient had difficulty getting up in the morning and worsening of dizziness, palpitations, and malaise during standing and walking, and orthostatic testing reproducibly induced tachycardia, lower-extremity tremor, marked sweating, and nausea. However, thyroid function tests revealed severe hyperthyroidism due to Graves’ disease. Following treatment, these symptoms resolved and repeat orthostatic testing after improvement of thyroid function no longer reproduced the symptoms or marked tachycardia observed during the initial test. Notably, propranolol had been discontinued two months before the repeat standing test; thus, the normalization of the heart rate response was observed in the absence of β-blocker treatment.

The marked orthostatic tachycardia in this patient also resembled POTS. However, the findings were not consistent with a diagnosis of primary POTS. Current international criteria for POTS in adolescents aged 12-19 years require a sustained increase in heart rate of ≥40 beats/min within 10 minutes of standing, orthostatic symptoms for at least three months, and the absence of other conditions that can explain sinus tachycardia, including hyperthyroidism [2]. In the present patient, the duration of symptoms was only one month, the resting heart rate was already elevated at 103-109 beats/min, and severe hyperthyroidism was present. Therefore, her presentation was considered more consistent with POTS-like orthostatic tachycardia associated with Graves’ disease than with primary POTS.

Lower-extremity tremor during orthostatic testing was another notable feature of this case. Tremor is observed in approximately 76% of patients with Graves’ disease and generally improves with normalization of thyroid function [7]. Graves’ disease-associated orthostatic tremor has also been reported, with lower-extremity tremor and unsteadiness occurring during standing and resolving after antithyroid treatment [8]. Although electromyography was not performed in our patient and orthostatic tremor therefore could not be definitively diagnosed, the emergence of lower-extremity tremor specifically during orthostatic stress and its disappearance following treatment suggest that hyperthyroidism contributed to this finding.

The overlap between Graves’ disease and OD or POTS-like orthostatic intolerance may extend beyond their clinical manifestations to the hemodynamic and autonomic effects of hyperthyroidism. In hyperthyroidism, excess thyroid hormone increases β-adrenergic receptor sensitivity, resulting in increased heart rate and cardiac output and decreased peripheral vascular resistance [9]. Furthermore, autonomic dysfunction characterized by increased sympathetic activity and decreased parasympathetic activity has been reported in patients with hyperthyroidism. The degree of autonomic dysfunction correlates with the severity of thyroid dysfunction and normalizes as thyroid function improves [9,10]. These hemodynamic and autonomic changes may contribute to the development of tachycardia and orthostatic intolerance during standing, thereby potentially causing OD-like and POTS-like symptoms. Although the possibility that Graves’ disease and an underlying orthostatic intolerance coincidentally coexisted in this patient cannot be completely excluded, the resolution of the orthostatic symptoms and abnormal responses after treatment supports the possibility that hyperthyroidism contributed substantially to her orthostatic presentation.

The active standing test is designed to assess symptoms and hemodynamic responses to orthostatic stress, and abnormal findings are not specific to OD or POTS. Therefore, possible underlying diseases may warrant consideration when interpreting the results of the active standing test. In particular, the baseline resting heart rate, in addition to the increase in heart rate after standing, may provide a clue to an underlying condition. In this patient, the resting heart rate was already elevated at 103-109 beats/min before standing, suggesting that the tachycardia was not solely related to orthostatic stress. Graves’ disease can present with a wide variety of symptoms and may be challenging to diagnose [11,12]. In patients presenting with OD-like symptoms accompanied by resting tachycardia, tremor, increased sweating, or weight loss, thyroid dysfunction may be considered in the differential diagnosis.

## Conclusions

Graves’ disease may present with OD-like symptoms and POTS-like orthostatic tachycardia during active standing testing. This case highlights the importance of considering underlying conditions, including thyroid dysfunction, when interpreting orthostatic symptoms and the results of the active standing test. Attention to the resting heart rate may help distinguish orthostatic tachycardia secondary to an underlying systemic condition from primary orthostatic intolerance. A careful review of accompanying systemic symptoms may also provide important diagnostic clues. Recognition of the underlying disorder may prevent unnecessary treatment directed solely at orthostatic symptoms.
